# Downregulation of the 5’-ectonucleotidase CD73 of CD8+ CTL of HIV infected patients correlates with immune activation and diminished IL-2 production

**DOI:** 10.1186/1742-4690-9-S2-P261

**Published:** 2012-09-13

**Authors:** I Toth, J Hauber, P Hartjen, J van Lunzen, J Schulze zur Wiesch

**Affiliations:** 1 Heinrich Pette Institute, Germany; 2 University of Hamburg, Hamburg, Germany

## Background

Chronic untreated HIV infection is immunologically characterized by irreversible loss of CD4+ T cells, general immune activation and CD4+ and CD8+ T cell dysfunction with diminished proliferative capacity. CD73 is an ectoenzyme (5'-ectonucleotidase) expressed on T cells converting 5'-AMP to adenosine, but there is additional evidence of ectonucleotidase-independent CD73 function. Altogether CD73 seems to play a role as a co-stimulatory molecule for T cell differentiation.

## Methods

Peripheral blood of a large cohort of 103 HIV infected patients at different stages of HIV disease, including long-term nonprogressors and elite controllers, was analyzed by multicolour flow cytometry to determine the expression of CD73+ on CD8+ CTL, CD4+ T effector cells and Tregs.

## Results

Surprisingly CD73 was not expressed on human regulatory T cells regardless of the infection status. However we find high expression of CD8+ in healthy controls. In HIV infection, CD73 seems to be generally suppressed on CD8+ T cells, independent of their naive or memory subtype. We find significant correlation between downregulation of CD73 and viremia, and there is an inverse correlation with CD8+ immune activation. Elite controllers show comparable CD73 expression to healthy controls. First proliferation studies show that that HIV-specific CD8+ CD73- T cell population produces less IL-2 than their CD73+ counterparts.

## Conclusion

CD73 is not expressed on human Tregs. CD73 downregulation of CD8+ T cells correlates with HIV disease progression. Further functional studies should look into the exact role of CD73 in HIV.

